# Overexpression of the Transcription Factor Azf1 Reveals Novel Regulatory Functions and Impacts β-Glucosidase Production in *Trichoderma reesei*

**DOI:** 10.3390/jof9121173

**Published:** 2023-12-07

**Authors:** David Batista Maués, Jhonatan Christian Maraschin, Diego Ângelo Duarte, Amanda Cristina Campos Antoniêto, Roberto N. Silva

**Affiliations:** Department of Biochemistry and Immunology, Ribeirão Preto Medical School, University of São Paulo, Ribeirão Preto 14049-900, SP, Brazil; dbmaues@gmail.com (D.B.M.); jhonatanmaraschin@gmail.com (J.C.M.); diegoaduarte@outlook.com (D.Â.D.); antonieto.acc@gmail.com (A.C.C.A.)

**Keywords:** *Trichoderma reesei*, cellulases, Azf1, transcription factor, overexpression, β-glucosidases

## Abstract

The fungus *Trichoderma reesei* is an essential producer of enzymes that degrade lignocellulosic biomass to produce value-added bioproducts. The cellulolytic system of *T. reesei* is controlled by several transcription factors (TFs) that efficiently regulate the production of these enzymes. Recently, a new TF named Azf1 was identified as a positive regulator of cellulase expression. Here, we investigated novel regulatory functions of Azf1 by its overexpression. In the mutant strain OEazf1, overexpression of *azf1* was achieved under both repression and induction conditions. Although *azf1* was more abundant in transcript and protein, overexpression of this TF did not activate transcription of the cellulase gene in the presence of the repressor glucose, suggesting that Azf1 may be subject to posttranslational regulation. In cellulose, the expression of *swo*, encoding the accessory protein swollenin, and the β-glucosidases *cel1a*, *cel1b*, *cel3b*, and *cel3g* increases in the early stages of cultivation. The increased production of these β-glucosidases increases the hydrolysis rate of cellobiose and sophorose, which activates carbon catabolite repression (CCR) and causes repression of cellulase genes and the regulator Xyr1 in the later stages of cultivation. Moreover, overexpression of *azf1* led to increased cellulase activity in *T. reesei* during long-term cultivation in cellulose and sugarcane bagasse. Our results provide new insights into the mechanisms regulating Azf1 and novel genes that are important targets of this TF. This work contributes to a better understanding of the complex mechanisms regulating cellulase expression in *T. reesei*. It will contribute to the development of strains with higher production of these essential enzymes.

## 1. Introduction

Lignocellulosic biomass (LCB) is the most abundant renewable carbon source on Earth, reaching a global production of up to 1.3 billion tons per year, which can be applied in the production of biofuels and fine chemicals [1]. Lignocellulose consists of three main components: cellulose (35–50%), hemicellulose (20–30%), and lignin (5–30%). Together, cellulose and hemicellulose constitute holocellulose, which is coated with lignin. LCB degradation is carried out by several enzymes, named cellulases, hemicellulases, and lignin-modifying enzymes, that act synergically to depolymerize lignocellulosic biomass and thus release fermentable sugars that can be used in the production of bioethanol and other value-added products [2].

The saprophytic fungus *Trichoderma reesei* is widely known for its high capacity to secrete cellulases, reaching a production of 100 g/L in industrial conditions [3]. Despite producing high amounts of these enzymes, the *T. reesei* cellulolytic system is one of the smallest and smartest among biomass-degrading fungi. Its genome encodes the cellobiohydrolases Cel7a and Cel6a, which act at the end of cellulose chains, releasing β-cellobiose; seven endoglucanases that cleave internal β-1,4-glucosidic bonds of cellulose chains, generating glucan chains of different sizes; and eleven β-glucosidases, which hydrolyze cellobiose producing glucose. In addition, it encodes 16 hemicellulases that act in the hemicellulose polymer, 6 lytic polysaccharide monooxygenases (LPMOs), enzymes that belong to the glycosyl hydrolase (GH) 61 family, and works on the degradation of lignocellulosic material through an oxidative mechanism; and also the accessory protein swollenin [4,5,6]. Together and synergically, these enzymes can efficiently degrade plant biomass into fermentable sugars.

The production of cellulases involves several processes, from sensing nutrients, signal transduction, gene expression, and translation to secretion of these proteins. The expression of plant cell wall-degrading enzymes is rigidly regulated by several transcription factors (TFs) and does not occur without an inducer. Oligosaccharides derived from lignocellulosic biomass, such as cellulose and xylan, are activators of the transcription of genes that encode holocellulolytic enzymes. In *T. reesei*, the most potent inducer of cellulase expression is the disaccharide sophorose, formed from cellobiose through a reaction called transglycosylation. In addition, lactose can also activate the expression of these enzymes, while glucose is the central repressor of cellulase genes [7,8].

The cellulolytic and hemicellulolytic genes are under the control of the zinc binuclear cluster protein Xyr1, the master activator of holocellulase expression. Xyr1 overexpression increases cellulase production, even without an inducer [9]. Another essential activator of cellulase expression is the transcription factor Ace3. Studies have shown that Ace3 mediates the expression of cellulases directly by binding to the promoters of cellulolytic genes [10]. Other positive regulators are Vib1, Rxe1, Clp1, Ace2, BglR and the HAP 2/3/5 complex [6,11,12].

The carbon catabolite repression (CCR) is a mechanism in which the fungus uses the most energetically favorable carbon source. The presence of glucose activates the CCR. In *T. reesei*, this mechanism is mediated by the C_2_H_2_ transcription factor Cre1. Cre1 is the central repressor of cellulase and hemicellulase expression. In addition, Cre1 represses the expression of the activators of cellulase expression, such as Xyr1 [13]. Also, Rce1 and Rce2 are cellulase-specific repressors that compete with Xyr1 and Ace3, respectively, for binding to the *cel7a* promoter [14,15]. Despite extensive studies, several TFs and different regulatory mechanisms that—in a coordinated way—regulate the expression of all the arsenal to degrade the plant cell wall are still unknown.

Recently, RNA-seq data analysis allowed us to identify a novel transcription factor named Azf1, homologous to Azf1p from *Saccharomyces cerevisiae*. In the budding yeast, Azf1p was initially identified as a protein that represses a mutation in the mitochondrial RNA polymerase gene *RPO41* [16]. Subsequently, Stein and coworkers showed that Azf1p is a predominantly nuclear protein and that its synthesis occurs preferentially under nonfermentable growth conditions and is almost undetectable in glucose [17]. Interestingly, Newcomb and his coauthors demonstrated that in glucose, Azf1p is involved in cell cycle regulation by activating transcription of *CLN3*, a G1 cyclin whose expression is directly related to the duration of the G1 phase of the cell cycle [18]. Finally, studies by Slattery and coauthors have shown that Azf1p has a function that depends on the carbon source: in glucose, Azf1p activates genes for carbon and energy metabolism, whereas in glycerol-lactate, it activates genes required for cell wall maintenance [19].

Functional characterization of Azf1 has shown that its role has been repurposed in *T. reesei.* While Azf1p plays an important role when yeast grows in the presence of glucose, expression of this regulator is almost undetectable in *T. reesei* in the presence of this sugar. Furthermore, Azf1 does not regulate the cell cycle in *T. reesei.* Deleting *azf1* decreases the expression of cellulolytic and hemicellulolytic genes in the presence of cellulose and sugarcane bagasse and impairs cellobiohydrolase and β-glucosidase activities. In addition, chromatin immunoprecipitation coupled with qPCR (ChIP-qPCR) showed that Azf1 directly regulates cellulase expression by binding to the promoters of the *cel7a*, *cel45a*, and *swo* genes [20].

Here, we obtained an overexpressed Azf1 strain (OEazf1) to obtain novel insights into its role in cellulase production. Our results show that the function of Azf1 in regulating cellulase expression is inducer-dependent, and some β-glucosidases are essential targets of this TF. The increase in the production of these β-glucosidases may be causing an early CCR that represses the expression of several cellulolytic genes. However, in long-term cultivation, *azf1* overexpression can bypass the CCR and increase the production of cellulases.

## 2. Materials and Methods

### 2.1. Strains and Culture Conditions

*Escherichia coli* DH5α was used for plasmid propagation. The strain was cultured on LB (Kasvi, Madrid, Spain) medium with or without ampicillin 100 µg/mL at 37 °C and 200 rpm.

*T. reesei* QM9414 (ATCC 26921) and the mutant OEazf1 strains were grown in MEX medium (3% (*w*/*v*) malt extract (Neogen, Neogen, MI, USA), 2% (*w*/*v*) agar (Kasvi, Madrid, Spain)) at 30 °C for 7–10 days until complete conidiation. For all experiments, 10^6^ conidia/mL for each strain were inoculated in Mandels–Andreotti medium [21] containing the respective carbon source, and the cultures were incubated in an orbital shaker (200 rpm) at 30 °C for the indicated time. In the experiments using mycelia as inoculum, 10^6^ conidia/mL for each strain were inoculated in Mandels–Andreotti medium containing 1% glycerol, and after 24 h (Figure S2), the mycelium was collected, washed with Mandels–Andreotti medium without a carbon source, and transferred to Mandels–Andreotti medium with 1% (*w*/*v*) cellulose (Synth, Diadema, SP, Brazil) or 1% (*w*/*v*) sugarcane bagasse. The experiments were conducted in triplicate for each sample. After induction, the mycelia were collected by filtration, frozen, and stored at −80 °C, and the supernatant was collected and stored at −20 °C.

### 2.2. Vector Construction and Fungal Transformation

The constitutive promoter *Pcdna1* was used to overexpress *azf1* [22,23]. The plasmid pLMcdna [24], which contains the hygromycin resistance gene (*hph*, encoding *E. coli* hygromycin B phosphotransferase) as a selection marker, was used as a backbone for constructing the vector. The cDNA sequence of *azf1* and part of the terminator region were synthesized by Genone Biotech (Rio de Janeiro, Brazil) and cloned into the plasmid pLMcdna, resulting in the vector pPcdna1-azf1(complete sequence in Figure S7). For protoplast-mediated transformation, 30 µg of pPcdna1-azf1 was used to transform strain QM9414 according to a protocol described previously [25]. Transformants were selected with hygromycin 50 µg/mL and confirmed by diagnostic PCR (Figure S1) using the primers listed in Table S1.

### 2.3. Gene Expression Analysis by RT-qPCR

*T. reesei* mycelia were macerated and the RNA was extracted using TRI^®^ (Sigma-Aldrich, St. Louis, MI, USA) according to the manufacturer’s instructions. For cDNA synthesis, 1 µg of RNA was first treated with DNAse I (Thermo Fisher Scientific, Waltham, MA, USA) to remove genomic DNA. After this step, cDNAs were synthesized using Maxima^TM^ first-strand cDNA synthesis (Thermo Fisher Scientific) according to the manufacturer’s instructions. They were diluted 50× and used for RT-qPCR analysis in Bio-Rad CFX96^TM^ equipment with SsoFast EvaGreen Supermix (Bio-Rad, Hercules, CA, USA) according to the manufacturer’s instructions. Gene expression levels were calculated from the threshold cycle according to the 2^−ΔCT^ method relative to transcript levels of β-actin [26]. The amplification program used in this study was as follows: 95 °C for 10 min followed by 39 cycles of 95 °C for 10 s and 60 °C for 30 s followed by a dissociation curve of 60 °C to 95 °C with an increment of 0.5 °C for 10 s per increment. The primers used for amplification of identified genes are described in Table S2. Statistical tests were performed using one-way ANOVA (and nonparametric testing), followed by Bonferroni’s test (to compare all pairs of columns) (available in Prism software v. 6.0) for comparing the gene expression levels of the wild-type (WT) QM9414 and mutant strains.

### 2.4. Protein and Enzyme Assays

Protein concentration was determined by the Bradford method using bovine serum albumin (BSA) (Sigma-Aldrich, St. Louis, MO, USA) as standard. To visualize the secretome, samples were precipitated using 10% tricarboxylic acid (TCA) (Sigma-Aldrich, St. Louis, MO, USA) and incubated in acetone at −20 °C overnight. The precipitated proteins were then pelleted by centrifugation at 10,000× *g* for 10 min at 4 °C. The pellets were washed three times in 0.07% β-mercaptoethanol and cold acetone, and then 30 µL of each sample was submitted to SDS-PAGE.

Carboxymethylcellulase (CMCase) activity was determined by adding 30 µL of 1% carboxymethyl cellulose (CMC) prepared in sodium acetate buffer (pH 4.8) and 30 µL of the sample. The reaction mixture was incubated at 50 °C for 30 min. Then, 60 µL of dinitrosalicyclic acid (DNS) was added to the reaction mixture, and the mixture was heated at 95 °C for 5 min. To analyze xylanase activity, 25 µL of the sample was incubated with 50 µL of 1% xylan and incubated at 50 °C for 30 min. Then, 75 µL of DNS was added to the reaction mixture and heated at 95 °C for 5 min. For both CMCase and xylanase activities, samples were read at an absorbance value of 540 nm. The β-glucosidase activity was determined by the addition of 50 µL of 50 mM sodium acetate buffer (pH 5.5), 10 µL of the sample, and 40 µL of 5 mM p-nitrophenyl (PNP)-glucoside substrate. The reaction mixture was incubated at 50 °C for 15 min, followed by adding 100 µL of 1 M sodium carbonate. For analysis of β-xylosidase activity, 50 µL of 50 mM sodium acetate (pH 4.8) was incubated with 10 µL of the sample and 40 µL of 5 mM PNP-xyloside. The reaction mixture was incubated at 50 °C for 15 min, followed by adding 100 µL of 1 M sodium carbonate. To analyze cellobiohydrolase activity, 50 µL of 50 mM sodium citrate (pH 4.8) was incubated with 10 µL of the sample and 40 µL of 5 mM PNP-cellobioside. The reaction mixture was incubated at 50 °C for 3 h 30 min and added to 100 µL of 1 M sodium carbonate. For analysis of β-glucosidase, β-xylosidase, and cellobiohydrolase activity, samples were read at an absorbance of 405 nm. One enzyme unit was defined as the amount of enzyme capable of releasing 1 µmol of reducing sugar or hydrolyzing 1 µmol of substrate per minute. Statistical tests were performed using one-way analysis of variance (ANOVA) followed by Bonferroni’s test (available in Prism software v. 6.0) for comparing the enzymatic activity levels of the WT and mutant strains. All the reagents were purchased from Sigma (Sigma-Aldrich, St. Louis, MO, USA).

### 2.5. Intracellular Protein Extraction

For protein extraction, the obtained frozen mycelia were ground to a fine powder using a mortar and pestle cooled in liquid nitrogen. Proteins were then extracted from the powdered samples (100 mg) using an extraction solution at pH 7.4 (0.8% NaCl, 0.02% KCl, 0.27% Na_2_HPO_4_·7H_2_O, 0.024% KH_2_PO_4_, 10 mM NaF, 1 mM Na_3_VO_4_, and 0.2% protease inhibitor cocktail (protease inhibitor mix 80-6501-23, GE Healthcare, Waukesha, WI, USA)). The samples were immediately sonicated in an ice bath (amplitude 60%, pulse 10 s on/10 s off, 1 min). Next, the sonicated mycelia were centrifuged twice at 4 °C and 14,000 rpm for 10 min, and the respective supernatants were carefully collected and stored at −20 °C in order to perform the subsequent analyses. The salts were purchased from Synth (Synth, Diadema, SP, Brazil).

### 2.6. Western Blot

Protein samples (30 µg) of extracted from mycelia of strains QM9414 and OEazf1 grown in glucose were subjected to 12% SDS-PAGE. Proteins were then transferred to a nitrocellulose membrane (GE Healthcare, Waukesha, WI, USA)) using a wet system (Trans-Blot^®^Turbo™, Bio-Rad, Hercules, CA, USA) with transfer buffer (48 mM Tris, 39 mM Glicina, 20% methanol, 0.0375% SDS). The membranes were blocked for 1 h at room temperature in Tris-buffered saline (TBS) containing 0.05% Tween (TBST) and 5% BSA. The membranes were then incubated overnight at 4 °C with an anti-Azf1 antibody (Rheabiotech, Campinas, SP, Brazil) diluted 1:3000 in the block solution. Membranes were washed three times for 10 min with TBST and incubated for 1 h with the appropriate peroxidase-conjugated anti-mouse secondary antibody. Membranes were again washed three times for 10 min each with TBST and revealed with ECL Prime (GE Healthcare) according to the manufacturer’s instructions. Finally, the ECL membranes were photographed using the ChemiDoc™ XRS+ (BioRad) photo documentation system.

The same membrane was incubated in a stripping buffer (100 mM β-mercaptoethanol, 2% SDS, 62.5 mM Tris-HCl) to remove the residual antibodies. It was then washed three times for 5 min with TBST and blocked for 1 h at room temperature with 5% skim milk in TBST. The membrane was then incubated for 2 h at room temperature with the anti-actin antibody diluted 1:20,000 in the blocking solution. The membrane was then washed three times for 5 min with TBST and incubated with the secondary anti-mouse antibody (1:5000 in TBST) for 1 h at room temperature, and the signal was detected as described previously. Densiometric analysis and quantification of the bands were performed using ImageJ software version 1.53 (https://imagej.nih.gov/ij/index.html, accessed on 18 October 2020).

### 2.7. Cellulase Production in Shake Flasks

The strains QM9414 and OEazf1 were grown in Mandels–Andreotti medium with glycerol 1% (*v*/*v*) for 24 h at 200 rpm and 30 °C. Then, the mycelia were washed with Mandels–Andreotti medium without a carbon source and transferred to the same medium containing 1% (wt/vol) of cellulose or sugarcane bagasse and cultivated for 96 h under the same conditions. Aliquots of 2 mL were collected every 24 h and centrifuged at 10,000× *g* for 15 min. The supernatant was then collected to measure enzyme activity as described above. The experiments were carried out in triplicate for each sample.

### 2.8. Bioinformatic Analysis

The putative Azf1-binding motif was obtained from the website http://zf.princeton.edu/index.php [27] (accessed on 30 August 2020) and had previously been validated by ChIP-qPCR [20]. Promoter sequences comprising 1 kb upstream of the ATG were obtained by an ad hoc script. The search for putative Azf1-binding sites in the promoter of the genes encoding the TFs differentially expressed in RNA-seq data (Table S3) was performed using FIMO [28], available on MEME [29], to identify possible Azf1 direct targets. Next, the putative binding motif of each one of these targets was generated. For TF *Tr4921*, encoding a zinc finger-type C_2_H_2_, the binding motif was generated as mentioned above for Azf1. For the others, which do not have the C_2_H_2_ domain, the amino acid sequence was used in a BLASTp search to identify orthologues in the *S. cerevisiae* genome, and the binding motif was obtained from the YeTFaSCo database [30] (accessed on 1 September 2020). The search for putative binding sites for those TFs in the promoter of the evaluated genes was performed in the same way as described above.

## 3. Results

### 3.1. Azf1-Mediated Cellulase Expression Is Inducer-Dependent

**Figure 1:**
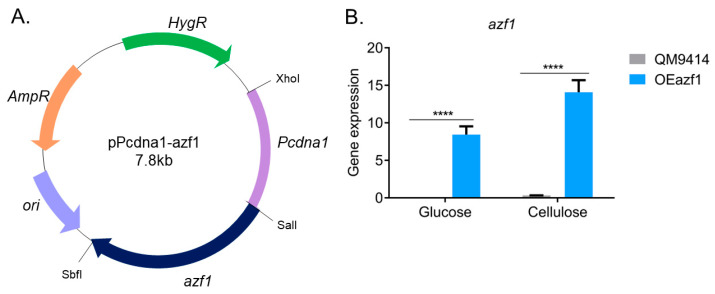
Overexpression of *azf1* in *T. reesei*. (**A**) The vector pPcdna1-azf1, where *azf1* is under the control of the constitutive promoter *Pcdna1*, and the hygromycin β phosphotransferase gene was used as the selection marker. (**B**) Gene expression of *azf1* in the parental strain QM9414 and mutant strain OEAzf1 cultivated for 24 h in glucose and cellulose (pregrown in glycerol for 24 h). Asterisks indicate significant differences (**** *p* ≤ 0.0001) as assessed by one-way ANOVA followed by Bonferroni’s test.

To overexpress *azf1* in *T. reesei*, we constructed a vector in which *azf1* is controlled by the constitute promoter from the *cdna1* gene (*Pcdna1*) [22,24]. The obtained vector, pPcdna1-azf1 (Figure 1A), was used to transform the *T. reesei* strain QM9414. One transformant, OEazf1, was selected for further analysis (Figure S1). First, we grew the WT QM9414 and mutant OEazf1 strains in Mandels–Andreotti medium containing the cellulase inducer source cellulose (after being grown in glycerol for 24 h) and glucose, a repressor of cellulase expression, and then analyzed the expression of *azf1* by RT-qPCR. As shown in Figure 1B, *azf1* is highly expressed in the OEazf1 strain, even in the repressor carbon source.

Next, we evaluated if overexpression of *azf1* can drive the expression of genes involved in biomass degradation in the presence of glucose. For analysis of gene expression, we selected direct targets of Azf1 previously validated by ChIP-qPCR (*cel7a*, *cel45a*, and *swo*), genes differentially expressed in RNA-seq (*xyn1*, *cel6a*, *cel3d* and the sugar transporter *lac1*) and RT-qPCR (*cel7b*, *cel61b* and *cel3b*) in experiments using the strains Δ*azf1* and TU6 [20].

**Figure 2:**
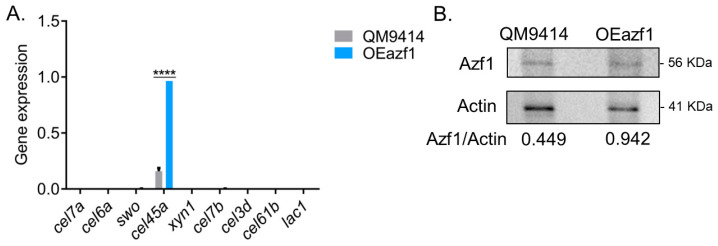
Overexpression of *azf1* cannot activate cellulase expression in the presence of glucose. (**A**) Expression profile of genes involved in biomass degradation in the QM9414 and OEazf1 strains grown in glucose for 24 h assessed by RT-qPCR. Asterisks indicate significant differences (**** *p* ≤ 0.0001) as evaluated by one-way ANOVA followed by Bonferroni’s test. (**B**). Western blot to detect Azf1 in the intracellular extracts from QM9414 and OEazf1 strains grown in glucose for 24 h. An anti-actin antibody was used to normalize the amount of protein in the membrane. Densiometric analysis and quantification of the bands were performed with the ImageJ software (https://imagej.nih.gov/ij/index.html, accessed on 18 October 2020).

In the presence of glucose, *azf1* overexpression increases only the transcript levels of the gene *cel45a*, which encodes endoglucanase and is a direct target of Azf1 [20]. Except for that, no difference was observed between the parental and mutant strains, with most evaluated genes showing virtually undetectable expression (Figure 2A). One hypothesis is that the azf1 transcript may be undergoing some posttranscriptional regulation and was not being translated. To confirm this assumption, Western blot was carried out using an anti-Azf1 antibody to detect the protein in the intracellular extract after growing the parental and mutant strains in glucose. It was possible to detect Azf1 in the extracts from both strains. However, Azf1 was more abundant in the OEazf1 mutant, increasing at least twofold (Figure 2B). Although more abundant in protein levels, Azf1 cannot activate cellulase gene expression in the presence of glucose. Taken together, these results suggest that the function of Azf1 in cellulase expression is inducer-dependent and is regulated preferentially in a posttranslational manner rather than at the transcriptional and protein level.

### 3.2. Azf1 Overexpression Decreases Cellulase Expression in the Presence of Cellulose

To understand the effects of Azf1 overexpression on producing holocellulases by *T. reesei* in inducing conditions, we first performed a transcriptional analysis by RT-qPCR. Two cultivations were performed: in the first, the WT and OEazf1 strains were pregrown in glycerol for 24 h and then transferred to cellulose, and the mycelium was collected after 8 and 24 h; in the second, the conidia of the WT and mutant strains were inoculated directly into cellulose and cultivated for 24 and 48 h. Gene expression was analyzed for the same genes analyzed in the culture on glucose.

**Figure 3:**
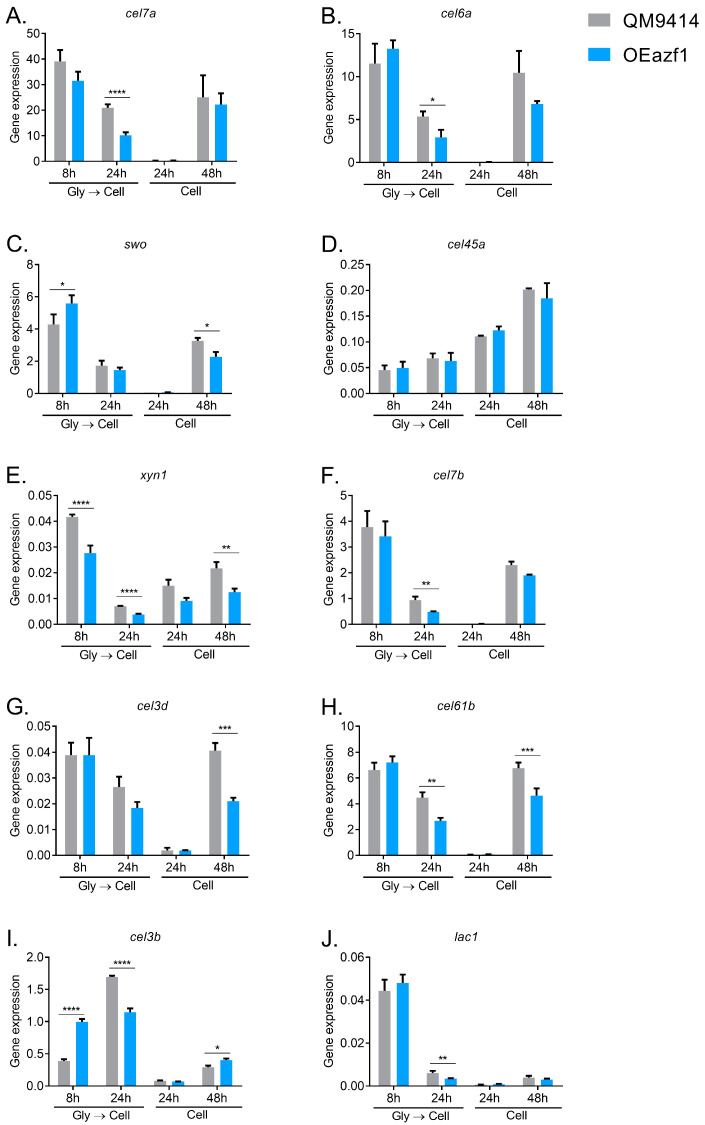
Overexpression of *azf1* decreases the expression of cellulolytic genes in the presence of cellulose. (**A**–**J**) Expression profile of genes involved in biomass degradation in the QM9414 and OEazf1 strains assessed by RT-qPCR. Strains were grown in cellulose for 8 or 24 h after being grown in glycerol for 24 h (Gly → Cell) or directly grown in cellulose for 24 or 48 h (Cell). Asterisks indicate significant differences (* *p* ≤ 0.05, ** *p* ≤ 0.01, *** *p* ≤ 0.001, **** *p* ≤ 0.0001) as assessed by one-way ANOVA followed by Bonferroni’s test.

In contrast to a previous report of Azf1 as a positive regulator of cellulase transcription [20], our results showed that the expression of most genes related to the biomass degradation evaluated was reduced in the OEazf1 strain (Figure 3). The genes for the accessory protein swollenin and β-glucosidase *cel3b* were the only ones that were shown to be more expressed in OEazf1 under any of the conditions analyzed (Figure 3C,I), and both genes are also repressed after 48 and 24 h of culture (pregrown in glycerol), respectively. The *swo* gene is directly regulated by Azf1, while *cel3b* presents possible binding sites for it [20]. Another direct target of Azf1, the main cellulase of *T. reesei*, *cel7a*, showed a reduction in expression in OEazf1 after 24 h of culture using mycelium as inoculum (Figure 3A). In contrast, *cel45a* showed no difference in expression between the WT and mutant strains (Figure 3D).

Interestingly, it is possible to observe that the reduction in gene expression occurs in the later stages of cultivation. When the fungus was pregrown in glycerol, after 24 h, *cel6a*, *cel7b*, *cel61b*, *cel3b*, and *lac1* were repressed in OEazf1 (Figure 3B,F,H–J), and when the conidia were used as an inoculum, after 48 h, it is possible to observe the repression of expression in the *swo*, *cel3d*, and *cel61b* genes (Figure 3C,G,H). In contrast, the *xyn1* gene is repressed in most analyzed conditions (Figure 3E), while after 8 h of cultivation, *azf1* overexpression increases the expression of *swo* and cel3b genes (Figure 3C,I). These results led us to hypothesize that the overexpression of Azf1 affects the production of cellulases in a temporal manner. In the early stages of cultivation, when the fungus recognizes the available carbon source—in this case, cellulose—overexpressed Azf1 activates the transcription of cellulase genes (*swo* and *cel3b*). However, during the course of cultivation, some regulation occurs that stands out from the overexpression of *azf1* and causes the repression of the expression of cellulases.

### 3.3. The Relationship between Azf1 Overexpression and Other Transcription Factors

**Figure 4:**
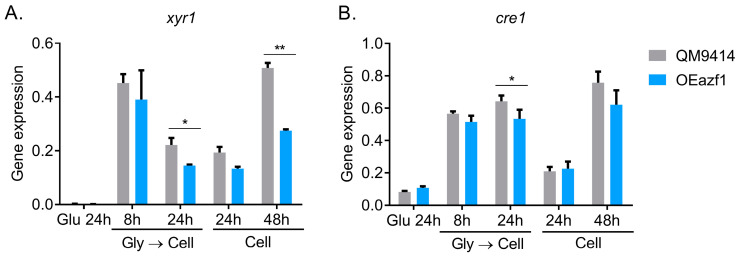
Overexpression of azf1 affects the expression of *xyr1*, the master regulator of cellulases in *T. reesei*. (**A**,**B**) Expression profile of *xyr1* (**A**) and *cre1* (**B**) TFs in QM9414 and OEazf1 strains assessed by RT-qPCR. Strains were grown in glucose for 24 h or cellulose for 8 or 24 h after being grown in glycerol for 24 h (Gly → Cell) or directly grown in cellulose for 24 or 48 h (Cell). Asterisks indicate significant differences (* *p* ≤ 0.05, ** *p* ≤ 0.01) as assessed by one-way ANOVA followed by Bonferroni’s test.

As Azf1 is a positive regulator of cellulase production in *T. reesei*, we concentrated on understanding why its overexpression caused the repression of cellulolytic genes in the most advanced stages of cultivation. In this way, we analyzed the behavior of the main transcription factors that regulate the production of cellulases in *T. reesei*: Xyr1 and Cre1. As shown in Figure 4A, Xyr1, the main activator of cellulase transcription, showed significantly reduced expression in the OEazf1 strain after 24 h of cultivation after being precultured in glycerol and 48 h when cultivated directly in cellulose. Interestingly, it was under these same conditions that the expression of most of the analyzed cellulase genes was repressed. Cre1 also showed a repressed expression in the OEazf1 strain in 24 h when the fungus was pregrown in glycerol (Figure 4B). These results suggest that overexpression of *azf1* triggers mechanisms that suppress *xyr1* transcription, which may be causing the repression of cellulase expression.

**Figure 5:**
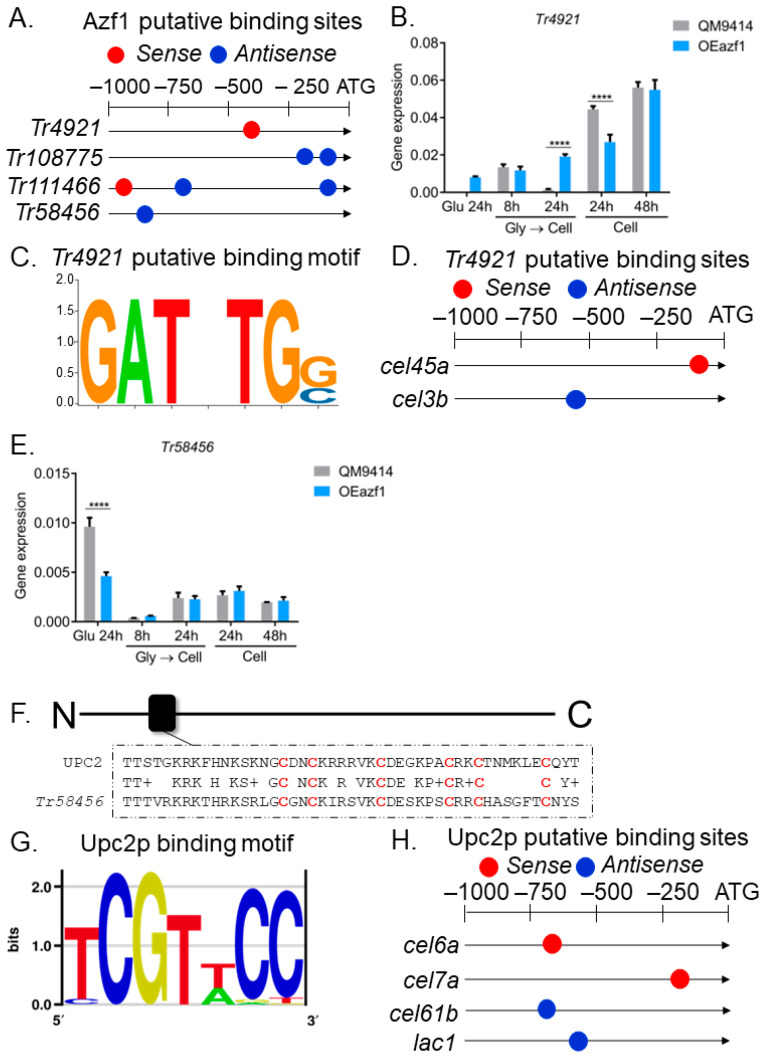
Regulatory network mediated by Azf1. (**A**) Putative Azf1-binding sites in the promoter of TFs differentially expressed in RNA-seq data. (**B**) Expression profile of *Tr4921* in QM9414 and OEazf1 strains assessed by RT-qPCR. (**C**) The putative binding motif of *Tr4921*. (**D**) Putative binding sites of *Tr4921* in the promoter of genes involved in biomass degradation analyzed by RT-qPCR. (**E**) Expression profile of *Tr58456* in QM9414 and OEazf1 strains analyzed by RT-qPCR. (**F**) Alignment between the sequences of *Tr58456* and its homologue in *S. cerevisiae* Upc2p in the DNA-binding domain (black box). Cysteine residues are highlighted in red. (**G**) Upc2p-binding motif. (**H**) Putative Upc2p-binding sites in the promoter of genes involved in biomass degradation analyzed by RT-qPCR. For gene expression analysis, strains were cultured in glucose for 24 h or in cellulose for 8 or 24 h after being grown in glycerol for 24 h (Gly → Cell) or directly in cellulose for 24 or 48 h (Cell). Asterisks indicate significant differences (**** *p* ≤ 0.0001) as determined by one-way ANOVA followed by Bonferroni’s test. Prediction of the putative binding motif for *Tr4921* and Upc2p and their putative binding sites in the promoters was performed by bioinformatic analysis (see Section 2.8).

Previous analysis of RNA-seq data obtained from the TU6 and Δ*azf1* strains grown in glycerol and sugarcane bagasse (SCB) identified eight differentially expressed transcription factors (Table S3) [20]. Using MEME, we searched for putative Azf1-binding sites on the promoters of these TFs. Our analysis showed that four TFs are possible direct targets of Azf1 (*p*-value ≤ 0.0001) (Figure 5A). Then, gene expression analysis was performed to assess whether the overexpression of *azf1* had any effect on the expression of these TFs (Figure 5B,E and Figure S3). The binding motif for these TFs was predicted by bioinformatic analysis and used to identify their possible targets among the genes analyzed with RT-qPCR.

The TF *Tr4921* presented a putative binding site for Azf1 in its promoter (Figure 5A), being upregulated by the latter in the RNA-seq data (Table S3) [20]. In glucose, it is possible to observe a slight increase in its expression in the OEazf1 strain, while in the QM9414 strain, its expression is practically nonexistent. Also consistent with the data obtained in the transcriptome, Azf1 overexpression significantly increased the expression of *Tr4921* in the cellulose culture after 24 h (pregrown in glycerol) (Figure 5B). When the conidia were used as inoculum, *Tr4921* expression at 24 h of culture was repressed in the OEazf1 strain, which could also be caused by other regulatory mechanisms in *T. reesei* (Figure 5B). A *Tr4921*-binding motif was predicted (Figure 5C), and this TF has putative binding sites in the promoters of the *cel45a* and *cel3b* genes (Figure 5D). Once Azf1 overexpression caused a slight increase in the expression of *Tr4921* in glucose, these results may indicate a possible genetic interaction between Azf1 and *Tr4921* to activate the expression of *cel45a* in the presence of glucose.

Regarding *Tr58456*, this TF was downregulated by Azf1 in RNA-seq data (Table S3) [20]. As shown in Figure 5E, this gene is repressed in the OEAzf1 strain in glucose. However, *azf1* overexpression did not affect the expression of *Tr58456* in cellulose (Figure 5E). This gene encodes a Zn_2_Cys_6_ zinc finger transcription factor, and we performed a search for homologues in the *S. cerevisiae* genome aiming to generate its putative binding motif. *Tr58456* is a homologue of transcription factor Upc2p (ID 30810) from *S. cerevisiae* (56.2% identity, e-value 2.35 × 10^−6^). Sequence alignment showed that the DNA-binding domain is highly conserved between the proteins (Figure 5F), so we proceeded with the analysis. The Upc2p-binding motif resolved by Gordan and coauthors [31] was obtained from the YeTFaSCo database (Figure 5G) and used in the search for putative binding sites in the promoters of the genes analyzed. Upc2p presents putative binding sites in the promoters of the *cel7a*, *cel6a*, *cel61b*, and *lac1* genes, all repressed in the OEazf1 strain (Figure 5H). As *Tr58456* was shown to be more expressed in glucose than cellulose and by the logic that Azf1 is a positive regulator of cellulase expression, *Tr58456* is likely to act as a repressor of cellulase production, repressed by Azf1 in glucose and sugarcane bagasse [20]. It was not observed that the overexpression of *azf1* suppresses the expression of *Tr58456* in cellulose. Therefore, the latter may be repressing the expression of the genes *cel7a*, *cel6a*, *cel61b*, and *lac1*. However, the functional characterization of *Tr4921* and *Tr58456* is necessary to understand their role in regulating cellulase expression.

Transcription factors *Tr108775* and *Tr111466* were downregulated by Azf1 in RNA-seq (Table S3). The overexpression of *azf1* significantly reduced the expression of *Tr108775* in one of the conditions analyzed (Figure S3B). However, *Tr111466* showed higher expression in the OEazf1 strain (Figure S3C). Again, other regulatory mechanisms may be activating the transcription of *Tr111466*, or the relationship of Azf1 and this TF in cellulose may be different from that observed in sugarcane bagasse, a more complex and heterogeneous carbon source, causing an increase in its expression. Unfortunately, it was impossible to generate the binding motif for these TFs, as they are not of the C_2_H_2_ type and do not have homologues in *S. cerevisiae*. Therefore, it is not possible to know whether they are targets among the cellulase genes evaluated. These results suggest that overexpression of *azf1* impacts the expression of other regulators that may be causing the phenotypes observed here.

### 3.4. Effects of Azf1 Overexpression on Cellulase Production

**Figure 6:**
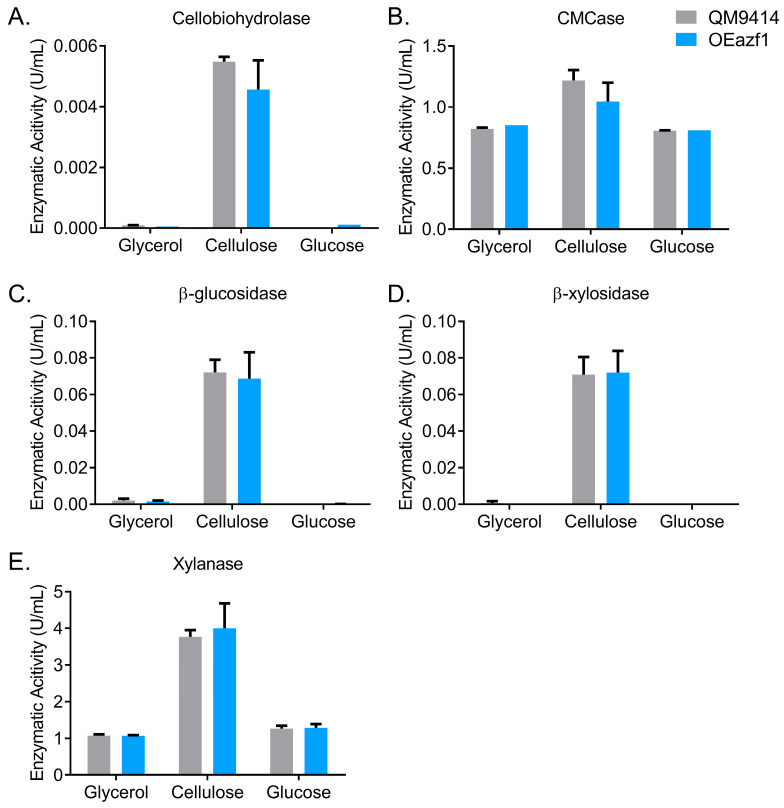
Overexpression of *azf1* does not affect cellulase production in the early cultivation phase. Enzymatic activities of QM9414 and OEazf1 strains grown in glycerol, cellulose (pregrown in glycerol), and glucose for 24 h. Supernatants were used to measure cellobiohydrolase (**A**), CMCase (**B**), β-glucosidase (**C**), β-xylosidase (**D**), and xylanase (**E**). The glucose culture supernatant was dialyzed. No significant differences were observed between strains QM9414 and OEazf1.

Next, the effects of Azf1 overexpression on cellulase production were evaluated. Supernatants of the QM9414 and OEazf1 strains grown in glucose, glycerol, and cellulose were used to measure cellobiohydrolase, CMCase, β-glucosidase, β-xylosidase, and xylanase activities. Figure 6 shows no enzymatic activity was detected in the glucose and glycerol cultivations for either strain, reinforcing that Azf1 overexpression does not increase cellulase production without an inducer. Interestingly, although the expression of most cellulases was reduced in the OEazf1 strain, the enzymatic activities showed no significant difference from the parental strain (Figure 6A–E). Protein determination in the supernatant and visualization of the secretome on SDS-PAGE confirmed no significant differences between the two strains (Figure S4). Therefore, the enzymatic activities do not reflect the repression of cellulase expression in the OEazf1 strain.

### 3.5. Azf1 Overexpression Impacts the Production of Intracellular β-Glucosidases

**Figure 7:**
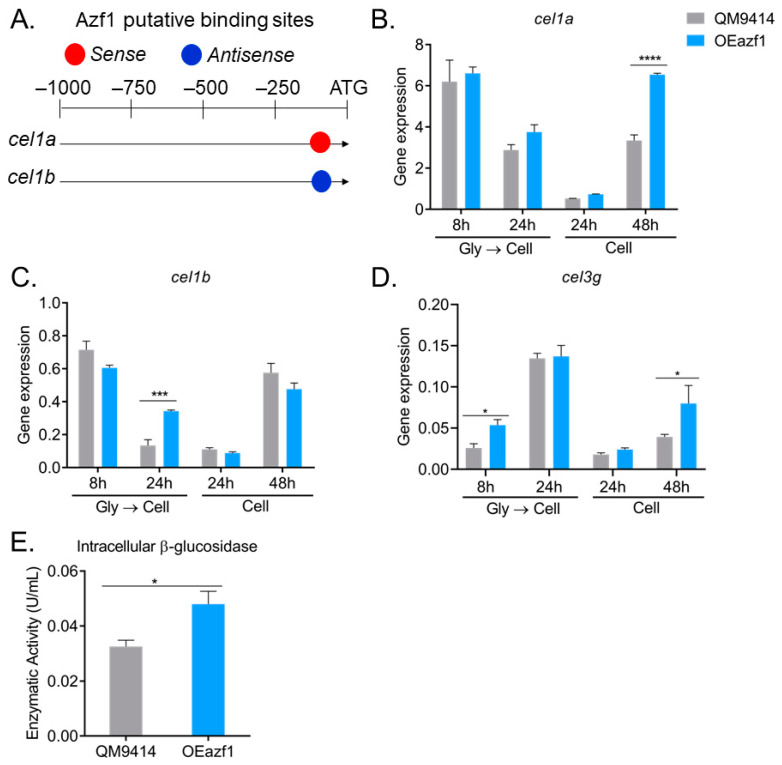
Overexpression of azf1 increases the production of intracellular β-glucosidases. (**A**) Azf1 putative binding sites in the promoter of *cel1a* and *cel1b* genes. (**B**–**D**) Expression profile of *cel1a* (**B**), *cel1b* (**C**), and *cel3g* (**D**) genes in QM9414 and OEazf1 strains assessed by RT-qPCR. Strains were grown in cellulose for 8 or 24 h after being grown in glycerol for 24 h (Gly → Cell) or directly grown in cellulose for 24 or 48 h (Cell). (**E**) β-glucosidase activity in the intracellular extracts from QM9414 and OEazf1 strains. Strains were grown in cellulose after being grown in glycerol for 24 h, and then 0.1 g of mycelium from each strain was used for protein extraction. Asterisks indicate significant differences (* *p* ≤ 0.05, *** *p* ≤ 0.001, **** *p* ≤ 0.0001) as assessed by one-way ANOVA followed by Bonferroni’s test.

The *T. reesei* genome encodes 11 β-glucosidases. The most studied are Cel3a/BglI, the extracellular β-glucosidase most produced by this fungus, and Cel1a and Cel1b, intracellular β-glucosidases [32]. These intracellular β-glucosidases play an essential role in regulating the expression of cellulases in different carbon sources, as they are responsible for hydrolyzing cellobiose that is transported into the cell [33]. As Azf1 has putative binding sites in the promoter of *cel1a* and *cel1b* genes (Figure 7A) [20], the expression of these genes in QM9414 and OEazf1 strains grown on cellulose was analyzed. Azf1 overexpression significantly increased the expression of *cel1a* and cel1b in the 48 h and 24 h cultures (pregrown in glycerol), respectively (Figure 7B,C).

Recently, the β-glucosidase Cel3g/Bgl3i was shown to be involved in cellulase production by *T. reesei*. This enzyme is also an intracellular β-glucosidase, and its deletion increases cellulase production by *T. reesei* [32]. Although we did not find a putative binding site for Azf1 in the *cel3g* promoter, gene expression analysis shows that overexpression of *azf1* positively affects the expression of *cel3g* after 8 h of cultivation in cellulose (after precultivation in glycerol) and after 48 h of cultivation when the fungus is directly inoculated in cellulose (Figure 7D). These results show that overexpression of *azf1* impacts the expression of important β-glucosidases in *T. reesei*.

To verify whether the increase in expression of these β-glucosidases is reflected in protein translation, we measured β-glucosidase activity in the intracellular extract of strains QM9414 and OEazf1 cultured in cellulose for 24 h (pregrown in glycerol). Consistent with the gene expression results, the OEazf1 strain showed significantly higher β-glucosidase activity in the intracellular extract than QM9414 (Figure 7E). Therefore, overexpression of *azf1* increases the production of intracellular β-glucosidases.

Interestingly, the increase in expression of *cel1a* and *cel1b* occurs under conditions where most cellulase genes are repressed: after 24 h when the fungus was precultured in glycerol and after 48 h when there was no preculture (Figure 3). Also, *cel3g* is highly expressed during the initial cultivation period, and this enzyme is thought to be responsible for sophorose hydrolysis [32]. Since these enzymes are responsible for the hydrolysis of cellobiose and sophorose in the intracellular environment, our results suggest that overexpression of *azf1* increases the production of these β-glucosidases, which increases the rate of cellobiose and sophorose hydrolysis in the cell and releases much glucose. The glucose present in the cell activates the CCR, which represses the expression of cellulases and xyr1, as observed via the transcription factor Cre1.

### 3.6. Azf1 Overexpression Increases Cellulase Production in Long-Term Cultivation

**Figure 8:**
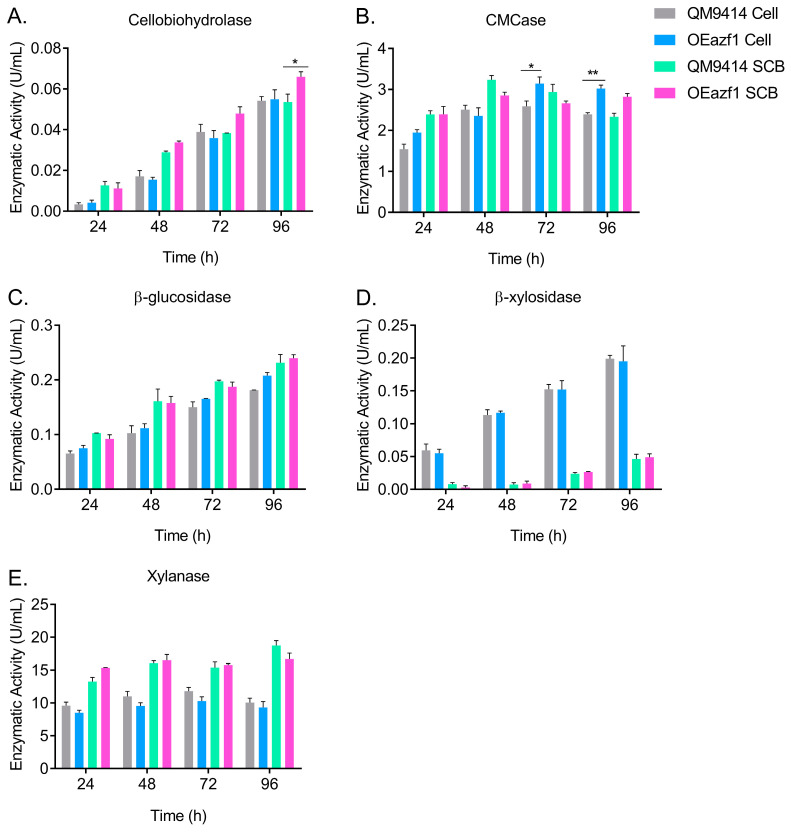
Overexpression of *azf1* increases cellulase production in the long cultivation phase. Enzymatic activities of QM9414 and OEazf1 strains grown in cellulose (Cell) or sugarcane bagasse (SCB) after being grown in glycerol for 24 h. Supernatants were collected every 24 h for 96 h and used to measure cellobiohydrolase (**A**), CMCase (**B**), β-glucosidase (**C**), β-xylosidase (**D**), and xylanase (**E**). Asterisks indicate significant differences (* *p* ≤ 0.05, ** *p* ≤ 0.01) as assessed by one-way ANOVA followed by Bonferroni’s test.

To better understand the effects of Azf1 overexpression on cellulase production by *T. reesei*, the strains QM9414 and OEazf1 were grown in cellulose or sugarcane bagasse for 96 h, and aliquots were taken every 24 h to measure enzymatic activity. In both carbon sources, the strains behaved similarly, with activity increasing every 24 h (Figure 8). However, in cellulose culture from 72 h, the OEAzf1 mutant showed higher values of CMCase activity (Figure 8B). In sugarcane bagasse, *azf1* overexpression caused an increase in cellobiohydrolase activity after 96 h of cultivation (Figure 8A). Therefore, these results indicate that in the long term, *azf1* overexpression can increase the production of cellulases by *T. reesei*.

## 4. Discussion

*T. reesei* has a complex and finely controlled cellulolytic system. The orchestrated regulation of this system allows the fungus to recognize available nutrients and capture them efficiently. Several regulatory mechanisms and transcription factors regulate holocellulase production by *T. reesei*. Among these regulators, we identified a new one, homologous to *S. cerevisiae* Azf1p, which presents high gene expression in the presence of plant cell wall derivatives, such as cellulose and sugarcane bagasse, acting as a positive regulator of the production of cellulases in *T. reesei* [20]. In the present work, we deeply investigated the role of Azf1 in regulating cellulase expression through its overexpression.

To overexpress *azf1*, we used the *Pcdna1* promoter, considered a strong promoter. Some cellulases under *Pcdna1* control were produced in large quantities when *T. reesei* was grown on glucose [22,24]. Furthermore, overexpression of the transcription factor *xyr1* using *Pcdna1* also increased cellulase production in cellulose culture [11]. Overexpression of *azf1* using this promoter was achieved under repressing and inducing conditions in the OEazf1 mutant strain (Figure 1B).

First, it was evaluated whether the overexpression of *azf1* could increase cellulase expression in the presence of glucose, a repressor carbon source. Although Azf1 was more abundant in the OEazf1 strain, both at the transcriptional and protein levels, its overexpression did not affect the transcription of cellulase genes, which was corroborated by the absence of enzyme activity (Figure 2). The *cel45a* endoglucanase was the only gene that showed a significant increase in expression in the mutant strain. These results suggest that Azf1 by itself cannot induce cellulase expression without an inducer. The same effects are observed in some biomass-degrading fungi. In *Penicillium oxalicum* and *Aspergillus nidulans*, for example, the overexpression of the transcription factor ClrB, the main regulator of cellulase expression in these fungi, did not induce cellulase expression in the absence of an inducer either [34,35].

TFs must alter the local chromatin to activate or repress transcription of their target genes. In *T. reesei*, chromatin remodeling plays an important role in regulating cellulase expression, and it is known that chromatin status depends on inducing or repressing conditions [36]. For example, nucleosome rearrangements in the promoter regions of the *cel7a* and *cel6a* genes are necessary for gene expression [37,38]. As in glucose, chromatin is more condensed in the regions of cellulase genes to repress gene expression. One hypothesis is that Azf1 cannot access gene promoters and thus initiate transcription. It is known that the main regulators of cellulase expression in *T. reesei*, Xyr1, and Cre1, are involved in chromatin remodeling [36,39]. Studies have shown that Xyr1 directly interacts with the TrSNF12 subunit of the SWI–SNF complex, a conserved ATP-dependent chromatin remodeler [40]. In addition, Xyr1 has an interdependent relationship with the Lae1 protein, a possible methyltransferase [41]. Recently, it was shown that the chromatin remodeler ISW1 has an essential role in cellulase expression unrelated to Xyr1, suggesting other TFs may interact with it to activate cellulase expression [42].

Our results also suggest that Azf1 undergoes carbon source-dependent transcriptional regulation and may also be the target of posttranslational regulations. Posttranslational modifications such as phosphorylation regulate protein function, protein–protein interaction, subcellular localization, etc. It is known that the phosphorylation of transcription factors Cre1 and Ace2 is essential for their DNA-binding activity [43,44]. In the Cre1 homologue in *A. nidulans*, CreA phosphorylation regulates its subcellular localization, DNA binding, and protein accumulation [45,46]. From the analysis of the amino acid sequence of Azf1 using the tools NetPhos (www.cbs.dtu.dk/services/NetPhos/) and NetworKIN (networkin.info) (both accessed on 15 October 2020), 81 phosphorylation sites were predicted, where the sites with the highest scores are for protein kinase C (PKC) (Figure S5). Therefore, posttranslational regulation may explain why Azf1 does not induce cellulase expression under repressing conditions.

Although Azf1 was previously identified as a positive regulator for cellulase expression in *T. reesei*, overexpression of this TF did not increase the expression of cellulolytic genes. On the contrary, *azf1* overexpression decreases the transcription of several cellulolytic genes after 24 h (pregrown in glycerol) or 48 h of cultivation in cellulose (Figure 3), in addition to a decrease in *xyr1* expression (Figure 4A). Interestingly, the measure of enzymatic activity in the supernatant showed no significant difference between the WT and mutant strains (Figure 6). This may indicate that Azf1 has a role in the posttranscriptional regulation of cellulase genes, and its overexpression increases events such as mRNA processing and/or protein translation/secretion. In *T. reesei*, the G protein-coupled receptor (GPCR) CSG1 is involved in posttranscriptional mechanisms of cellulase genes in the presence of cellulose and lactose [47]. In another cellulolytic fungus, *Neurospora crassa*, the β subunit of the G protein CPC-2 also plays a role in the posttranscriptional regulation of cellulolytic genes, as does the enzyme adenylate cyclase and its cAMP product [48].

As Azf1 is a positive regulator of cellulase production in *T. reesei*, we focused on understanding why its overexpression caused the repression of cellulolytic genes. In addition to affecting the expression of *xyr1* and *cre1* (Figure 4), *azf1* overexpression impacts a regulatory network involving diverse other TFs that may be direct targets of Azf1. Except for the *Tr111466* gene, all TFs had their expression modulated consistently with the data obtained from RNA-seq under all the conditions analyzed (Figure 5 and Figure S4). The transcription factor *Tr4921* has possible binding sites in the promoter region of the *cel45a* and *cel3b* genes (Figure 5D). The overexpression of *azf1* slightly increased the expression of *Tr4921* in glucose (Figure 5B), where it is also possible to observe a significant increase in the expression of *cel45a* (Figure 2A). These results suggest that Azf1 and *Tr4921* can jointly activate the expression of this cellulase under repressor conditions.

*Tr58456* is homologous to the *S. cerevisiae* Upc2p transcription factor (Figure 5F), which has possible binding sites in the promoters of the *cel7a*, *cel6a*, *cel61b*, and *lac1* genes (Figure 5H). The overexpression of *azf1* represses the expression of *Tr58456* in glucose, but does not affect its expression in cellulose (Figure 5E). Since Azf1 is a positive regulator, it makes more sense that it suppresses negative regulators. In addition to the fact that *Tr58456* is more expressed in glucose, we assume that the latter acts as a repressor of cellulase expression and may be responsible for reducing the expression of *cel7a*, *cel6a*, *cel61b*, and *lac1*. Interestingly, Upc2p participates in the sterol regulatory element (SRE)-binding proteins (SREBP) pathway, which regulates sterol biosynthesis [49]. In fungi, in addition to regulating sterol homeostasis, the SREBP pathway is involved in adaptation to hypoxia, drug resistance, and virulence [50]. In *N. crassa* and *T. reesei*, the SREBP pathway acts as a negative regulator of protein secretion under lignocellulolytic conditions [51]. An in-depth investigation of this pathway in *N. crassa* has shown that it represses the expression of LPMOs, which require molecular oxygen for catalytic activity [52]. Interestingly, LMPO *cel61b* has a reduced expression (Figure 3H), and our analysis showed that it has possible sites for Upc2p (Figure 5H). However, further characterization of the SREBP pathway in *T. reesei*, as well as the transcription factor *Tr58456*, is needed to understand their roles in cellulase expression and secretion.

As intracellular β-glucosidases play an important role in inducing cellulase expression [33] and Azf1 possibly directly regulates Cel1a and Cel1b enzymes (Figure 7A) [20], we investigated whether the overexpression of *azf1* affects the production of intracellular β-glucosidases in *T. reesei*. The *cel1a*, *cel1b*, and *cel3g* genes are significantly more expressed in the OEazf1 strain (Figure 7B–D), which caused an increase in intracellular β-glucosidase activity (Figure 7E). The *cel1a* and *cel1b* genes are more expressed under the same conditions in which the cellulase genes and the transcription factor *xyr1* were repressed: 24 h, when there was pregrowth in glycerol, and 48 h, when conidia were used as inoculum. And *cel3g* is more expressed at the initial cultivation phase (8 h). Our results suggest that the increased production of intracellular β-glucosidases may have caused the repression of the cellulase expression we observed.

The β-glucosidases Cel1a and Cel1b play an important role in the production of cellulases in *T. reesei*, as they regulate the cellobiose hydrolysis rate. Deleting these genes causes a delay in the induction of cellulase production in the presence of cellulose and lactose, but not in sophorose or cellobiose [53,54]. Functional characterization of Cel3g showed that these β-glucosidases are critical in cellulase induction and may be the principal enzymes responsible for sophorose hydrolysis in the cellular interior [32]. Therefore, we believe that high levels of *cel1a*, *cel1b*, and *cel3g* in the OEazf1 strain increase the rate of cellobiose and sophorose hydrolysis. Glucose resulting from this hydrolysis activates CCR, and Cre1 moves to the nucleus and represses the cellulase and the activator *xyr1* genes [55]. Recently, Pang and coauthors investigated the role of all 11 β-glucosidases in cellulase production by overexpressing them in the RutC30 strain. Overexpression of *cel1a* and *cel1b* were the ones that most reduced cellulase production. Also, their results indicate that Cel1a and Cel3g are secreted in the extracellular medium, even under the control of their native promoter [56]. This inconsistency with the data obtained previously [32,54] can be explained by the relatively poor secretion of these enzymes in the early stages of cultivation, being detected in the extracellular extract after 96 h [56].

Regarding Cel1b, an in-depth investigation of its role in regulating cellulase expression was also carried out by Pang and coauthors. This β-glucosidase is in fact intracellular, and its overexpression causes a dramatic reduction in cellulase production [56,57]. However, this effect is not caused by early activation of CCR, but because this enzyme affects the expression of sugar transporters that are crucial for cellulase signaling (such as Crt1) and causes endoplasmic reticulum (ER) deregulation [57,58,59]. We investigated the expression profile of some genes involved in ER stress (Figure S6). Overexpression of *azf1* causes a decrease in the expression of the transcription factor *hac1*, which regulates the unfolded protein response (UPR) (Figure S6A), an increase in the expression of *hrd1*, an E3 ubiquitin ligase that mediates the degradation of folding-defective proteins in the ER lumen, participates in the ER degradation association (ERAD) pathway (Figure S6D), and causes a minor effect in the expression of the molecular chaperone *bip1* (Figure S6B) [60]. These results suggest that perhaps increased expression of *azf1* and *cel1b* affects similar cellular processes in *T. reesei*.

**Figure 9:**
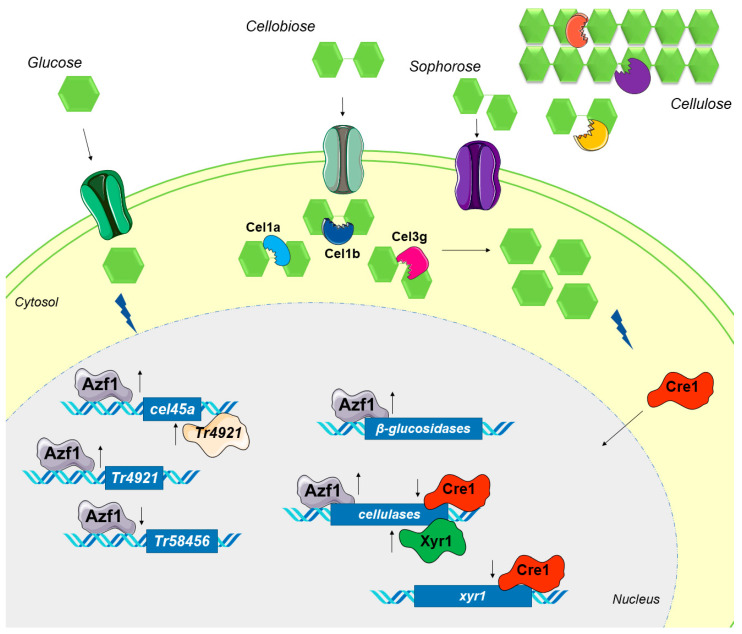
Hypothetical model of regulatory mechanisms caused by overexpression of *azf1.* In glucose, overexpressed Azf1 activates (up arrow) the expression of the cellulase *cel45a* and the transcription factor *Tr4921* and represses (down arrow) the expression of *Tr58456*. *Tr4921* can also activate the transcription of *cel45a*. When grown in the presence of cellulose, the synergistic action of endo- and exoglucanases releases cellobiose, which is transported into the cytosol. In addition, intracellular and extracellular β-glucosidases can produce sophorose from cellobiose. Azf1 increases the expression of cellulases, especially the intracellular β-glucosidases *cel1a*, *cel1b*, and *cel3g*, which hydrolyze cellobiose and sophorose, releasing more glucose into the cell environment. The resulting glucose activates CCR, and Cre1 migrates to the nucleus, where it represses the expression of cellulases and the key activator *xyr1*.

In the study by Pang and coworkers, *cel1b* overexpression did not increase the intracellular β-glucosidase activity [57], but when *azf1* is overexpressed, this increase is observed. As such, in our hypothesis, when *azf1* is overexpressed, the dose and synergism of Cel1a, Cel1b, Cel3g, and other β-glucosidases could be causing this early CCR. A hypothetical model of the regulatory mechanisms caused by the overexpression of *azf1* in *T. reesei* is presented in Figure 9.

Interestingly, the hyper-cellulolytic *T. reesei* strain PC-3-7 possesses a mutation in the *cel1a* gene, which produces an enzyme with a reduced cellobiose metabolic rate, causing an alleviation in CCR, which improves the production of cellulases [61]. Furthermore, the deletion of the transcription factor BglR, an activator of the expression of β-glucosidases, results in increased production of cellulases due to the gradual transport of cellobiose into the intracellular medium [33]. In *N. crassa*, deletion of the three main β-glucosidase genes induces the production of cellulases in the presence of cellobiose, and this induction restores, both at transcriptional and protein levels, the wild-type response to cellulose [62]. These results demonstrate the importance of intracellular β-glucosidases in the production of cellulases in different organisms.

The initial goal of this work was to generate a *T. reesei* strain with a high level of cellulase production. Therefore, we investigated the effects of *azf1* overexpression in long-term cultivation. It seems that after a long period of cultivation, *azf1* overexpression can bypass the CCR and have a positive impact on cellulase production by *T. reesei*. After 72 h of cultivation in cellulose, there is an increase in CMCase activity in the OEazf1 strain. In sugarcane bagasse, there is an increase in cellobiohydrolase activity after 96 h of cultivation in the OEazf1 strain. However, the activities of β-glucosidase, β-xylosidase, and xylanase remained similar in both strains. Therefore, these results show that the overexpression of *azf1* increases the production of cellulases.

## 5. Conclusions

In conclusion, new roles of the TF Azf1 were revealed through its overexpression. Our results suggest that the role of Azf1 in cellulase expression is inducer-dependent, and its overexpression temporally affects cellulase production. In addition, the β-glucosidases Cel1a, Cel1b, and Cel3g are important targets of Azf1. Finally, overexpression of *azf1* can increase cellulase production when it overcomes CCR. These results contribute to a better understanding of the molecular mechanisms involved in the complex regulation of cellulase gene expression in *T. reesei*.

## Data Availability

All relevant data are within the paper and its Supporting Information files. Strains and plasmids are available upon request.

## Supplementary Materials

The following supporting information can be downloaded at https://www.mdpi.com/article/10.3390/jof9121173/s1. Table S1: Primers used in PCR verification of transformants; Table S2: Primers used in gene expression analysis by RT-qPCR; Table S3: Transcription factors differentially expressed in RNA-seq; Figure S1: PCR verification of transformants from QM9414; Figure S2: Dry weight of the strains; Figure S3: Regulatory network mediated by Azf1; Figure S4: Protein secretion by QM9414 and OEazf1 strains; Figure S5: Prediction of phosphorylation sites in Azf1; Figure S6: Overexpression of *azf1* affects the expression of genes involved in ER stress. Figure S7: Sequence of the vector pPcdna1-azf1.
