# Supplementary material for: Overexpression of the Transcription Factor Azf1 Reveals Novel Regulatory Functions and Impacts β-Glucosidase Production in Trichoderma reesei
Source: J Fungi (Basel). 2023 Dec 7;9(12):1173. doi: 10.3390/jof9121173 (PMC10744372; doi:10.3390/jof9121173)
Supplement: Supplementary file 1 [file jof-09-01173-s001.zip › jof-2731531-supplementary.pdf]

Supplementary Information

Overexpression of the transcription factor Azf1 reveals novel regulatory functions and impacts β-glucosidase production in *Trichoderma reesei*

Maués et al

Table S1

Primers used in PCR verification of transformants

Primer	Sequence (5’-3’)	Amplicon (bp)
ScCdnaAzfFw	CCCGACTTTGACATCATGAGGG	1103
ScCdnaAzfRv	CCGCTTTGTGTACGAAGTACG	
ScreeningHphF	CTGAACTCACCGCGACGTC	857
ScreeningHphR	GCCCAAGCTGCATCATC	

Table S2

Primers used in gene expression analysis by RT-qPCR

Gene	***Foward*** **(5'-3')**	***Reverse*** **(5'-3')**
azf1	AGAATCTCACCATACGCCATG	CAATGCCCATGTGAAAGACG
cel7a	CCGAGCTTGGTAGTTACTCTG	GGTAGCCTTCTTGACTGAGT
cel6a	ACAAGAATGCATCGTCTCCG	TGTTCCACCCGTTGTAGTTG
swo	GAGTGAATGTCTTGATGG	CCAAACTATACGAGTAGCC
cel45a	CAGCGACGTCTACATTGG	TGGTCCAGAATGCACTCG
xyn1	GGCCAAATTATCGTCAACTGTC	TCTGTCTTTTGGGCTTGGAG
cel7b	CCCTCAACACTAGCCACCAG	AGGTCTTGGAGGTGTCAACG
cel3d	AACCCAGCATATCTCAACTACG	CTTGAAGGTAGCGTAAGACAGG
cel61b	ACTATGTCTTCCGCCATGAAC	CACGCACTGAGGATAGTTCTG
lac1	CACAACCCTCTCTATGCTGAT	GGGCACGATATTTGGGATATG
xyr1	CAATCCTCTCCGTCGCTATTC	CTGTTGCCGAATGTGTTGAC
cre1	CTCCTACTCGTCCTTTGTCATG	GCAAGCATCGTAATGTCGTTG
cel1a	TTTGCCTGGTCGCTCATG	AATCAGCTCGTCAAACAGCG
cel3b	CCAGGATAACTTCAACGA GGG	ATGTGGAGGTTGGAGAACTTG
cel1b	CCATCTACATCACCGAGAACG	TCCAAGTGCGAGTCAAAGTAG
cel3g	CCCCAAGACACATACACAGAG	TTTGTGTAGCTCAGACCGTG
Tr4921	GTATTCTGGATATTACCGGCCTC	GTTGTGTTGAGACTTGAAGGC
Tr111466	AGAGAATAGAGACCCCTTACCG	AGAAAAGAGGATAAGCCGAGTG
Tr58456	CAATGTCCTCTACTTCCTCAGC	ATCACAAGAGTATGCCAGTATAGC
Tr108775	CAACAGCAATGACAGCATCG	AAGTGAAAGCATCGGAGGTC
hac1	ACGAATCACACTCCACATCC	TGCTCCTTTTCATCTTCCGTC
bip1	TCACCATCACCAACGACAAG	GAAGGCGTAGTTCTCAAGACC
pdi1	AGAACCCCGTCACCTACAG	TGTACTTGCCGTTCTCCTTG
hrd1	GAAACCCCTCCACCTGAAAG	CATCCTCAGCCTCTTCCATG
actin	TGAGAGCGGTGGTATCCACG	GGTACCACCAGACATGACAATGTT

Table S3

Transcription factors differentially expressed in RNA-Seq.

The genes that presented *p*-value ≤ 0.05 and the difference (DE) between the sugarcane bagasse/glycerol fold change (FC) (in log2) of the parental and mutant strains higher than 1 or lower than -1 were considered differentially expressed. In addition, a second analysis was performed, where genes that presented fold change (FC) Δ*azf1*/TU6 (in log2) higher than 1 or lower than -1 were also considered differentially expressed[1].

FC: log2 *Fold change.* SCB: sugarcarne bagasse. G: glycerol. DE: FC TU6 - FC Δ*azf1*.

ID	Description	FC TU6 (SCB/G)	FC ***Δazf1*** (SCB/G)	DE
122448	C2H2 transcription factor	1.846302326	0.653485176	1.19281715
4921	C2H2 transcriptional regulator	1.41039561	0.355420805	1.054974805
108775	transcription factor AbaA	1.850519158	3.005474359	-1.15495520
58456	Zn2Cys6 transcriptional regulator	0.854156382	2.153289836	-1.29913345
112539	Zn2Cys6 transcriptional regulator	3.3283038	4.645091627	-1.31678782
106677	Zn2Cys6 transcriptional regulator	-0.846807122	0.470111248	-1.31691837
54007	transcriptional regulator HMGtype	2.938849044	5.478157963	-2.53930891
		**FC (*Δazf1*/TU6)**		
111466	Zn2Cys6 transcriptional regulator	2.471271		

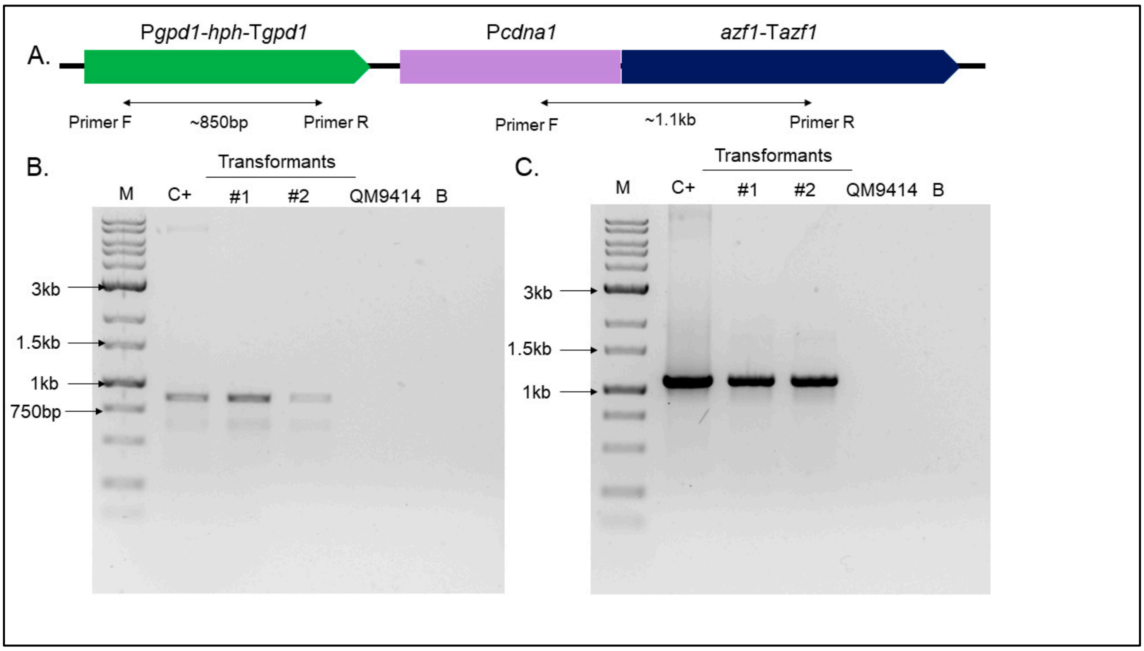

Figure S1. PCR verification of transformants from QM9414. (A) Schematic representation of verification strategy of the transformants. (B) Amplification of *hph* gene. (C) Amplification of part of the Pcdna1-azf1 cassette. C+: positive control (plasmid pPcdna1-azf1). B: blank, no DNA. M: Molecular weight marker.

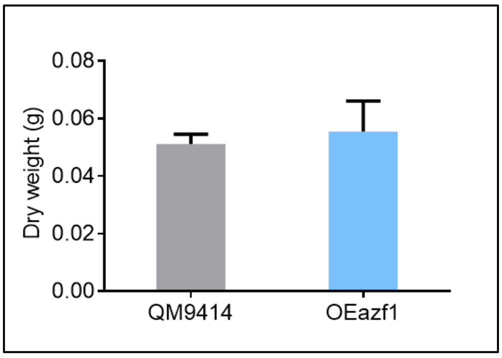

Figure S2. Dry weight of the strains. Mycelia dry weight of QM9414 and OEazf1 strains. Strains were grown in glycerol 1% for 24h and then the mycelium was filtered by vacuum and dried at 70ºC for 6h and the weight was determined. The experiments were performed in triplicate for each sample. No significant difference was observed between the strains.

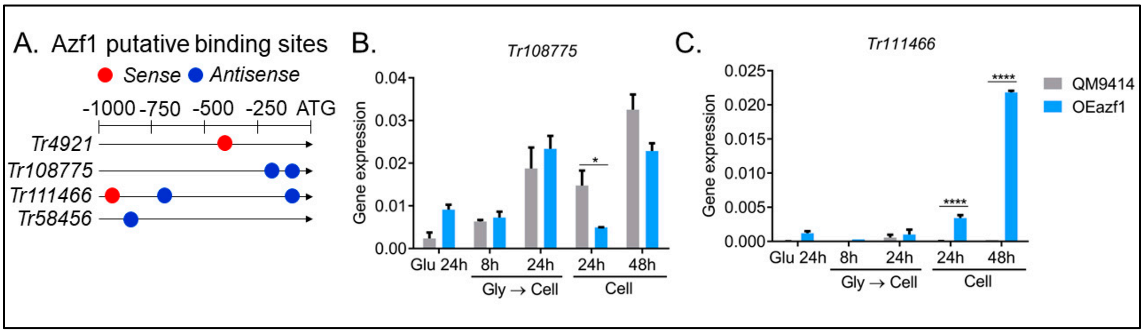

Figure S3. Regulatoy network mediated by Azf1. (A) Azf1 putative binding sites in the promoter of TFs diferentially expressed in RNA-Seq data. (B-C) Expression profile of *Tr108775* (B) and *Tr111466* (C) in QM9414 and OEazf1 strains assessed by RT-qPCR. Strains were grown in glucose for 24 h or in cellulose for 8 or 24h after pregrown in glycerol for 24 h (Gly → Cell) or directly grown in cellulose for 24 or 48 h (Cell). Asterisks indicate significant differences (*p ≤ 0.05, ****p ≤ 0.0001) as assessed by One-way ANOVA followed by Bonferroni's test.

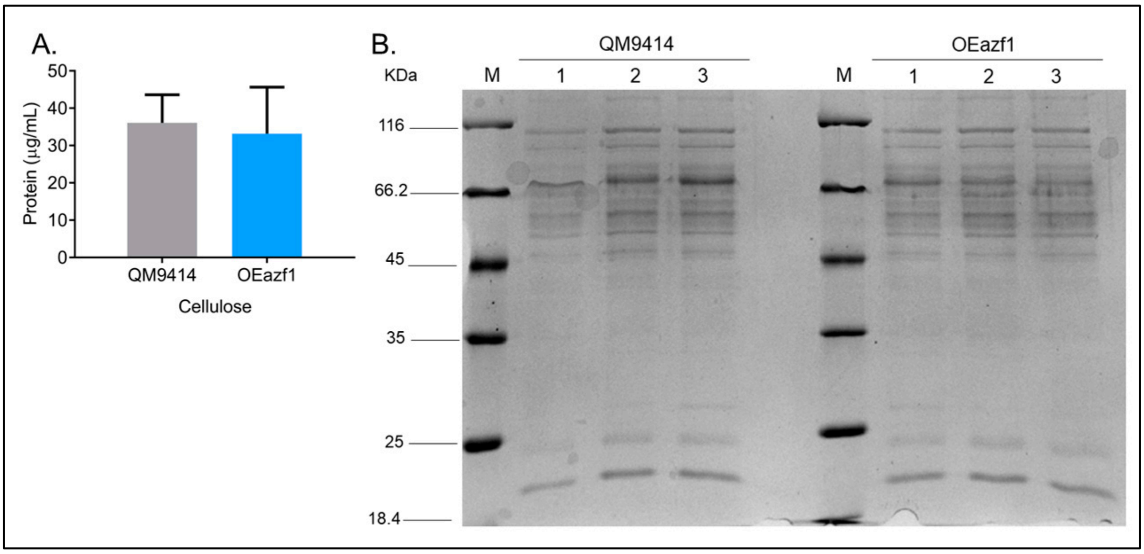

Figure S4. Protein secretion by QM9414 and OEazf1 strains. (A) Protein concentration in the supernatant of the strains QM9414 and OEazf1 grown on cellulose for 24h after pre-grown in glycerol for 24h. (B) SDS-Page for visualization of the secretome of strains QM9414 and OEazf1 grown in cellulose.

```
MALAAQQHTQADWGRWSHQIPQSFPMMGSPGFMSYDPRAQDGSQMQRQVS    #    50
AQYLVNSNYNQPPMPTASSPQYQHAGPFSYVPYHSPPPSTPLGSPFKSEF    #   100
PEHPLTRMTHSTVDRHHSQAMRDYQPYSPVSRRGSISSVATKPSAAPVTP    #   150
GPTTPGSFTSSSDAQSPSTPNPQTASQPVSSKTLTYNETVHPGDRISFRT    #   200
DVDELMKAIQKTQTTDECQQTLTPARTPKNCTTSTPVLRTQSGKPRKQWV    #   250
CDGPNCGKAFVQKTHRDIHRRTHTGHRPYVCTMENCGLTFSQRGNLKTHI    #   300
RRHTGEKPFSCAACGKCFAQRGNLRSHEETHKGLKPFVCRLDDCNKSFSQ    #   350
LGNMKTHQNNFHKETLQKLTHMFVQFSENGEVPRDYQDLFEYFQKHYKNS    #   400
NKGVKGRGKTRAVAARGPQDSAFRQAASPVPALLKTPATTHLPQMTMPAH    #   450
DPHGRISPYAMTQGAANTLSNVLRNPNPSYGLYGPTFAPGPVRDGVFHMG    #   500
IASHLS
```

Figure S5. Prediction of phosphorylation sites in Azf1. The putative phosphorylation sites are highlighted in red. The prediction was performed using the tools NetPhos (www.cbs.dtu.dk/services/NetPhos/) and NetworKIN (networkin.info), using the amino acid sequence of Azf1. The sites with the highest scores are for Protein kinase C (PkC).

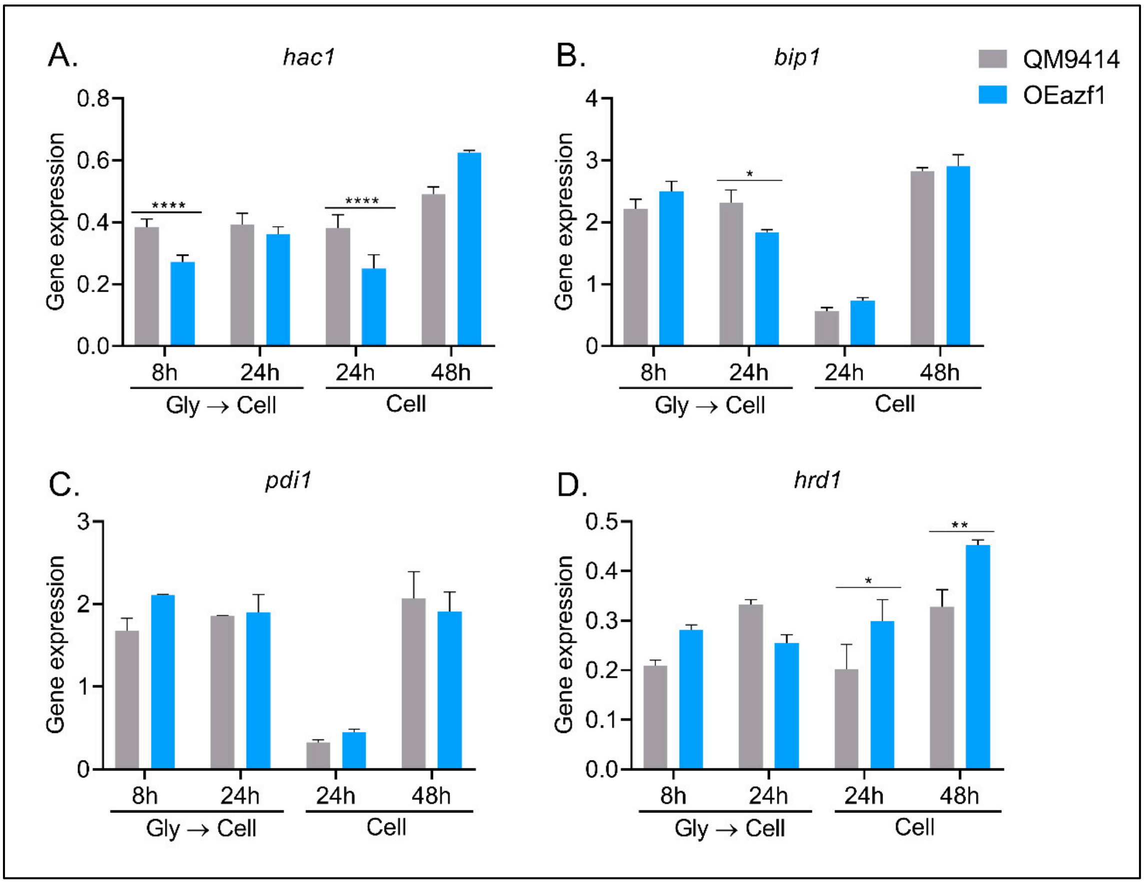

Figure S6. Overexpression of *azf1* affects the expression of genes involved in ER stress. (A-D) Expression profile of genes involved in ER stress *hac1* (A), *bip1* (B), *pdi1* (C) and *hrd1* (D). Strains were grown in cellulose for 8 or 24 h after pregrown in glycerol for 24 h (Gly → Cell) or directly grown in cellulose for 24 or 48 h (Cell). Asterisks indicate significant differences (*$p \leq 0.05$, **$p \leq 0.01$, ****$p \leq 0.0001$) as assessed by One-way ANOVA followed by Bonferroni's test.

tcgcgcgtttcggtgatgacggtgaaaacctctgacacatgcagctcccggagacggtcacagcttgtctgtaagcggatgccgg
gagcagacaagcccgtcagggcgcgtcagcgggtgttggcgggtgtcggggctggcttaactatgcggcatcagagcagattgt
actgagagtgcaccatatgcggtgtgaaataccgcacagatgcgtaaggagaaaataccgcatcaggcgccattcgccattcag
gctgcgcaactgttgggaagggcgatcggtgcgggcctcttcgctattacgccagctggcgaaagggggatgtgctgcaaggcg
attaagttgggtaacgccagggttttcccagtcacgacgttgtaaaacgacggccagtgaattcgagctcggtacccggggatccg
agagctaccttacatcaatatggccagcacctcttcggcgatacatactcgccaccccagccggggcgattgtgtgtactaggtag
gctcgtactataccagcaggagaggtgctgcttggcaatcgtgctcagctgttaggttgtacttgtatggtacttgtaaggtggtcatgc
agttgctaaggtacctagggagggattcaacgagccctgcttccaatgtccatctggataggatggcggctggcggggccgaag
ctgggaactcgccaacagtcatatgtaatagctcaagttgatgataccgttttgccaggattaggatgcgagaagcagcatgaatgt
cgctcatccgatgccgcatcaccgttgtgtcagaaacgaccaagctaagcaactaaggtaccttaccgtccactatctcaggtaac
caggtactaccagctaccctacctgccgtgcctacctgctttagtattaatctttccacctccctcctcaatcttcttttccctcctctcctctt
tttttttcttcctcctcttcttctccataaccattcctaacaacatcgacattctctcctaatcaccagcctcgcaaatcctcaggttagtatt
actactactacaatcatcaccacgatgctccgcccgacgatgcggcttctgttcgcctgcccctcctctcactcgtgcccttgacgag
ctaccccgccagactctcctgcgtcaccaatttttttccctatttacccctcctccctctctccctctcgtttcttcctaacaaacaaccacc
accaaaatctctttggaagctcacgactcacgcaagctcaattcgcagatacaaatctagaatgaaaaagcctgaactcaccgc
gacgtctgtcgagaagtttctgatcgaaaagttcgacagcgtctccgacctgatgcagctctcggagggcgaagaatctcgtgcttt
cagcttcgatgtaggagggcgtggatatgtcctgcgggtaaatagctgcgccgatggtttctacaaagatcgttatgtttatcggcact
ttgcatcggccgcgctcccgattccggaagtgcttgacattggggaattcagcgagagcctgacctattgcatctcccgccgtgcac
agggtgtcacgttgcaagacctgcctgaaaccgaactgcccgctgttctgcagccggtcgcggaggccatggatgcgatcgctgc
ggccgatcttagccagacgagcgggttcggcccattcggaccgcaaggaatcggtcaatacactacatggcgtgatttcatatgc
gcgattgctgatccccatgtgtatcactggcaaactgtgatggacgacaccgtcagtgcgtccgtcgcgcaggctctcgatgagct
gatgctttgggccgaggactgccccgaagtccggcacctcgtgcacgcggatttcggctccaacaatgtcctgacggacaatggc
cgcataacagcggtcattgactggagcgaggcgatgttcggggattcccaatacgaggtcgccaacatcttcttctggaggccgtg
gttggcttgtatggagcagcagacgcgctacttcgagcggaggcatccggagcttgcaggatcgccgcggctccgggcgtatatg
ctccgcattggtcttgaccaactctatcagagcttggttgacggcaatttcgatgatgcagcttgggcgcagggtcgatgcgacgca
atcgtccgatccggagccgggactgtcgggcgtacacaaatcgcccgcagaagcgcggccgtctggaccgatggctgtgtaga
agtactcgccgatagtggaaaccgacgccccagcactcgtccgagggcaaaggaataatgcatgtgctgtgttcctcagaatgg
gccccagaagggcgtcgagcattgtctatgaatgcaaacaaaaatagtaaataaatagtaattctggccatgacgaatagagcc
aatctgctccacttgactatccttgtgactgtatcgtatgtcgaacccttgactgcccattcaaacaattgtaaaggaatatgagctaca
agttatgtctcacgtttgcgtgcgagcccgtttgtacgttattttgagaaagcgttgccatcacatgctcacagtcacttggcttacgatc
atgtttgcgatctttcggtaagaatacacagagtaacgattatacatccatcgctttctatgattaggtactcagacaacacatgggaa
acaagataaccatcgcatgcaaggtcgattccaatcatgatctggactggggtattccatctaagccatagtaccctcgagcagac
aatgatggtagcagcgcatggaagaacccggttgttcggaatgtccttgtgctaacagtggcatgattttacgttgcggctcatctcg
ccttggcaccggacctcagcaaatcttgtcacaacagcaatctcaaacagcctcatggttcccagattccctgattcagaactctag
agcggcagatgtcaaacgattctgacctagtaccttgagcatccctttcggatccggcccatgttctgcctgcccttctgagcacagc
aaacagcccaaaaggcgccggccgattcctttcccgggatgctccggagtggcaccacctcccaaaacaagcaaccttgaacc
cccccccaaatcaactgaagcgctcttcgcctaaccagcataagcccccccccaggatcgttaggccaagtggtagggccagc
caattagcgagcggccatttggaggtcatgggcgcagaatgtcctgacagtggtatgatattgactgcccggtgtgtgtggcatctg
gccataatcgcaggctgaggcgaggaagtctcgtgaggatgtcccgactttgacatcatgagggagtgagaaactgaagagaa
ggaaagcttcgaaggttcgataagggatgatttgcatggcgggcgacaggatgcgatggctcgttgggatacataatgcttgggtt

ggaagcgattccaggtcgtcttttttggttcatcatcacagcatcaacaagcaacgatacaagcaatccactgaggattacctctca
actcaaccactttccaaaccatctcaactccctaagattctttcagtgtattatcactaggatttttcccaagccggcttcaaaacacac
agataaaccaccaactctacaaccaaagactttttgatcaatccaacaacttctctcatcgatgtcgacatggccctcgcagctcaa
cagcatactcaggccgattggggccgctggtctcaccagattccacagagttttcccatgatgggctctccaggattcatgtcatacg
atcccagagctcaggacggcagtcagatgcagcgtcaggtgtctgctcagtacctggtgaactcgaactacaaccagcccccga
tgcccactgcttcgtctccccagtatcaacacgcagggccattttcctatgtgccttaccacagcccgccgccgtccactcctcttggtt
ccccattcaagagcgaatttcccgagcaccctcttacgcgcatgacacactctacggtcgatcgacaccattctcaggccatgagg
gactaccaaccttattctcctgtatcgaggaggggatcgatttcttcagtcgccaccaagccctcagcagctcccgtcacaccaggt
ccaactactcctggctctttcacttcaagttccgacgcccagagccccagcactccaaacccccagactgcgtctcagcctgtcag
ctccaagactctcacttacaatgagaccgttcatccgggcgataggatcagcttcagaaccgatgttgatgaactcatgaaggcca
tccagaagacacagacgaccgacgagtgtcagcaaacactcacacctgcgcgaacaccaaagaactgtaccacaagtactc
ccgtacttcgtacacaaagcgggaagccgagaaaacagtgggtttgcgatggccccaactgcggcaaggcctttgtccagaag
acgcatcgcgacattcaccgacgcactcacaccggccatcgaccatacgtctgcaccatggaaaattgcggtcttacgttctcgca
gcgaggaaacctcaagactcacatacgacgccacacaggtgaaaagccgttctcttgcgctgcttgtggcaagtgcttcgctcag
cgtgggaatcttcgatcccacgaggagacacacaaaggcctgaagcccttcgtctgccggctcgatgattgcaacaagtcgttttc
tcagctgggcaatatgaagactcatcagaacaactttcacaaagaaacgctccagaaactcacacacatgtttgtgcaattctcgg
agaacggcgaggtgcccagagactatcaggatcttttcgaatacttccagaagcactacaagaatagcaacaagggagtcaag
ggccgaggaaagactcgcgctgtggcagctcgtgggcctcaagattccgcgtttcggcaggctgcctccccagtgcccgcgttac
tgaagacgccggctacgactcatttgccccagatgacaatgccagcccatgatccccatggcagaatctcaccatacgccatga
cccagggagctgcgaacactctgagcaatgtcctgcgcaaccccaacccctcttacggcctttatggacccacgtttgccccggg
ccctgtacgagatggcgtctttcacatgggcattgcgagccacctatcctgagcgtcatcgacggcttggcttctccattggtattacg
atgcgtccaggatgcaaaagcttcacgaacccgacgacttcaagggtcgggacgctcaaaacacagtattcgtacacggacac
gaaaacgaaagtcgagatcacaccttccgaaagggacacaacatggctttgccatcagaagaagaagagaaaggggaaag
aagtacacgacgtatgtaacttgggcggtttattctgattcgatcatctgctgtgatcgagttcaaggcgaactcgacgcatcgcttgt
aaaagatgggtgaggtgtttttgtcggaggcctgcaggcatgcaagcttggcgtaatcatggtcatagctgtttcctgtgtgaaattgtt
atccgctcacaattccacacaacatacgagccggaagcataaagtgtaaagcctggggtgcctaatgagtgagctaactcacatt
aattgcgttgcgctcactgcccgctttccagtcgggaaacctgtcgtgccagctgcattaatgaatcggccaacgcgcggggaga
ggcggtttgcgtattgggcgctcttccgcttcctcgctcactgactcgctgcgctcggtcgttcggctgcggcgagcggtatcagctca
ctcaaaggcggtaatacggttatccacagaatcaggggataacgcaggaaagaacatgtgagcaaaaggccagcaaaaggc
caggaaccgtaaaaaggccgcgttgctggcgtttttccataggctccgcccccctgacgagcatcacaaaaatcgacgctcaagt
cagaggtggcgaaacccgacaggactataaagataccaggcgtttccccctggaagctccctcgtgcgctctcctgttccgaccc
tgccgcttaccggatacctgtccgcctttctcccttcgggaagcgtggcgctttctcatagctcacgctgtaggtatctcagttcggtgta
ggtcgttcgctccaagctgggctgtgtgcacgaaccccccgttcagcccgaccgctgcgccttatccggtaactatcgtcttgagtcc
aacccggtaagacacgacttatcgccactggcagcagccactggtaacaggattagcagagcgaggtatgtaggcggtgctac
agagttcttgaagtggtggcctaactacggctacactagaaggacagtatttggtatctgcgctctgctgaagccagttaccttcgga
aaaagagttggtagctcttgatccggcaaacaaaccaccgctggtagcggtggtttttttgtttgcaagcagcagattacgcgcaga
aaaaaaggatctcaagaagatcctttgatcttttctacggggtctgacgctcagtggaacgaaaactcacgttaagggattttggtca
tgagattatcaaaaaggatcttcacctagatccttttaaattaaaaatgaagttttaaatcaatctaaagtatatatgagtaaacttggtc
tgacagttaccaatgcttaatcagtgaggcacctatctcagcgatctgtctatttcgttcatccatagttgcctgactccccgtcgtgtag
ataactacgatacgggagggcttaccatctggccccagtgctgcaatgataccgcgagacccacgctcaccggctccagatttat

cagcaataaaccagccagccggaagggccgagcgcagaagtggtcctgcaactttatccgcctccatccagtctattaattgttgc
cgggaagctagagtaagtagttcgccagttaatagtttgcgcaacgttgttgccattgctacaggcatcgtggtgtcacgctcgtcgtt
tggtatggcttcattcagctccggttcccaacgatcaaggcgagttacatgatcccccatgttgtgcaaaaaagcggttagctccttc
ggtcctccgatcgttgtcagaagtaagttggccgcagtgttatcactcatggttatggcagcactgcataattctcttactgtcatgccat
ccgtaagatgcttttctgtgactggtgagtactcaaccaagtcattctgagaatagtgtatgcggcgaccgagttgctcttgcccggcg
tcaatacgggataataccgcgccacatagcagaactttaaaagtgctcatcattggaaaacgttcttcggggcgaaaactctcaa
ggatcttaccgctgttgagatccagttcgatgtaacccactcgtgcacccaactgatcttcagcatcttttactttcaccagcgtttctgg
gtgagcaaaaacaggaaggcaaaatgccgcaaaaaagggaataagggcgacacggaaatgttgaatactcatactcttccttt
ttcaatattattgaagcatttatcagggttattgtctcatgagcggatacatatttgaatgtatttagaaaaataaacaaataggggttcc
gcgcacatttccccgaaaagtgccacctgacgtctaagaaaccattattatcatgacattaacctataaaaataggcgtatcacga
ggccctttcgtc

Figure S7. Sequence of the vector pPcdna1-azf1. Nucleotides from 2771 to 3704 bp represent the Pcdna1 promoter and nucleotides from 3713 to 5233 represent the *azf1* cDNA sequence.
